# Part two: an unblinded, parallel, randomized study to assess nicotine pharmacokinetics of four Vuse Solo ENDS flavors in smokers

**DOI:** 10.1038/s41598-023-35439-3

**Published:** 2023-06-01

**Authors:** Brian M. Keyser, Kyung Soo Hong, Patricia DeLuca, Tao Jin, Bobbette A. Jones, Paul Nelson, Eckhardt Schmidt, Elaine K. Round

**Affiliations:** RAI Services Company, 401 N. Main Street, Winston-Salem, NC 27101 USA

**Keywords:** Biochemistry, Physiology, Biomarkers, Medical research

## Abstract

We report the findings from a randomized, parallel study designed to evaluate nicotine pharmacokinetics (PK) following 10 min of ad libitum use of electronic nicotine delivery system (ENDS) in four flavor variants. Subjects were randomized an investigational product (IP) and blood samples were collected for PK assessments during a test session. Primary endpoints were baseline-adjusted values of maximum plasma nicotine concentration (C_max_) and area under the nicotine concentration-vs-time curve up to 60 min (AUC_nic0–60_). Baseline-adjusted mean C_max_ ranged from 6.53 to 8.21 ng/mL, and mean AUC_nic0–60_ ranged from 206.87 to 263.52 ng min/mL for all ENDS IPs. Results of geometric mean C_max_ and AUC_nic0–60_ values were within 95% confidence intervals (CI) among the ENDS IP flavor variants tested.

## Introduction

Cigarette smoking is a leading cause of preventable premature death, and significantly increases the risk of developing lung cancer, heart disease, chronic bronchitis, chronic obstructive pulmonary disease and other serious diseases and adverse health conditions^1^. Whereas smoking conventional cigarettes requires combustion of tobacco, use of electronic nicotine delivery systems (ENDS) does not. ENDS were developed as potential reduced-harm alternative products for cigarette smokers. ENDS heat a nicotine-containing solution (e-liquid), which results in the generation of an aerosol containing fewer and lower levels of toxicants than are found in cigarette smoke^2,3^. This has been shown to reduce toxicant exposure to consumers who switch from cigarettes to ENDS^4–9^. Several public health authorities, such as Public Health England, Royal College of Physicians, and National Academies of Sciences, Engineering, and Medicine, have recognized the potential public health benefit of current smokers switching to ENDS^10–12^. A review by the National Academies of Sciences, Engineering and Medicine (NAS) concluded that “[t]he evidences about harm reduction suggests that across a range of studies and outcomes, e-cigarettes pose less risk to an individual than combustible cigarettes”^10^.

This publication is part of a three-part series describing the clinical assessment of Vuse Solo (cig-a-like ENDS). Vuse Solo has a rechargeable battery and has separate nicotine containing cartridges. The clinical studies included in this publication series describe the nicotine pharmacokinetics (PK) of Vuse Solo across four e-liquid flavors; an assessment of the abuse liability of Vuse Solo as compared to combustible cigarettes (CC) and a nicotine replacement therapy product^13^  and a study to assess whether use of Vuse Solo results in a reduction in exposure to harmful and potentially harmful constituents (HPHCs) after smokers are switched to the product for 5 days^14^.

We report findings from a clinical study that was performed as part of a regulatory submission to the FDA CTP to assess the nicotine PK parameters of Vuse Solo flavor variants after a single ad libitum use in solus and dual users of cigarette and ENDS. The study data was reviewed by the FDA and granted a marketing order for Original (tobacco) flavor Vuse Solo ENDS cartridges and the Vuse Solo power unit as they deemed the product was appropriate for the protection of public health^15^. The flavor variants in this study included the Original (tobacco flavor), Mint, Tropical, and Fusion. The study was executed following a parallel-group study design in which subjects were randomized to a single product, and nicotine PK was evaluated during and after a 10 min ad libitum product use during a test session, following a week of ambulatory product acclimation (at-home).

## Results

### Study population

A total of 148 subjects were enrolled and randomized; all subjects were randomized to one of four Vuse Solo investigational products (IPs) and 122 (82.4%) subjects completed all scheduled PK assessments. A total of 26 randomized subjects (17.6%) withdrew or early termed from the study for reasons unrelated to study products. Of these 122 study completers, four subjects were excluded from the final PK analysis due to C_max_ less than 1.0 ng/mL, suggesting the subjects did not gain familiarity with IP to use it appropriately during a test session. Thus, 118 (96.7% of study completers) subjects completed the study with evaluable PK data. The number of subjects who completed the study (and the associated percentage of completers with evaluable PK data) in each IP group are as follows: Original (n = 30, 93.3%), Mint (n = 34, 100%), Tropical (n = 29, 96.5%), and Fusion (n = 29, 96.5%).

The demographic and baseline characteristics of the subjects are summarized in Supplementary Table S1. Subjects were non-Hispanic (74.3%) and a higher number of males (55.4%) were recruited. The mean age was 33.7 years. Subjects enrolled in the study included 135 smokers (91.2%) and 13 dual smokers and ENDS users (8.7%). Subjects reported a mean smoking duration of 18 years with a mean of 16 cigarettes per day.

### Product use

ENDS product use, were determined by a difference in cartridge weights before and after a week of at-home trial period and after a test session were used as a surrogate for product use. The product use periods included a 1-week ambulatory (at-home) trial period and one 10-min ad libitum ENDS use period per PK test session. The differences between initial and final cartridge weights were calculated for both periods and results are shown in Table 1.

**Table 1:** Vuse Solo e-liquid usage (grams) during the at-home trial and PK assessment periods.

	Original	Mint	Tropical	Fusion
At-home trial period				
Number of subjects	35	39	38	36
Mean (SD)	0.2101 (0.2517)	0.1643 (0.1728)	0.2082 (0.2339)	0.2010 (0.2296)
Median (range)	0.1029 (0–0.8062)	0.1127 (0–0.7862)	0.0912 (0–0.7154)	0.1117 (0–0.7360)
Day 2 PK assessment				
Number of subjects	30	34	29	29
Mean (SD)	0.0348 (0.0269)	0.0318 (0.0171)	0.0371 (0.0229)	0.0357 (0.0270)
Median (range)	0.0266 (0.0003–0.1120)	0.0293 (0.0097–0.0882)	0.0374 (0.0076–0.1052)	0.02980 (0.0002–0.1420)

*SD* standard deviation.

### Pharmacokinetic results

**Figure 1:**
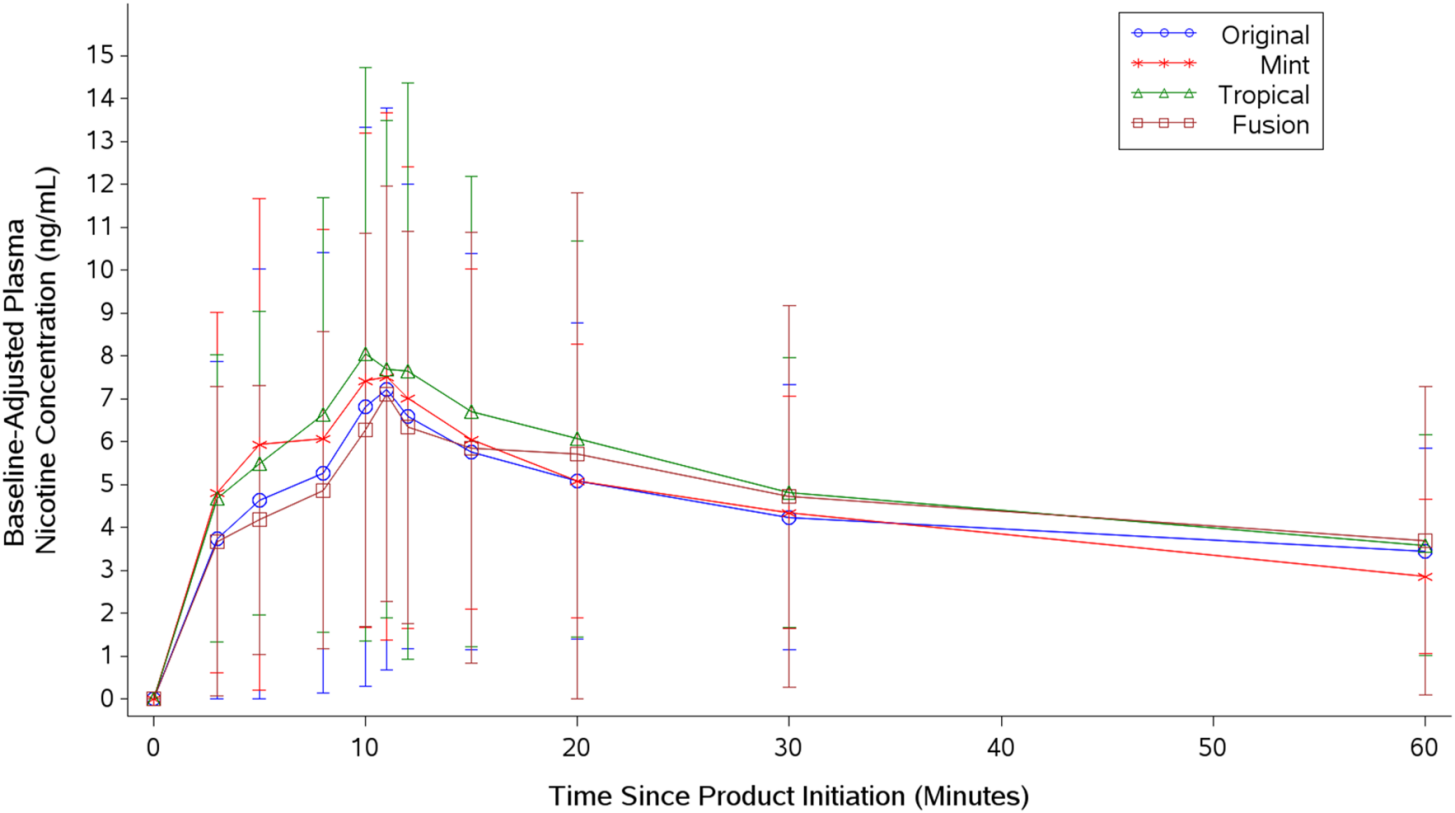
Baseline-adjusted plot of arithmetic mean plasma nicotine concentrations (0–60 min) for four flavor variants of Vuse Solo.

As illustrated in Fig. 1, baseline-adjusted plasma nicotine concentrations increased rapidly within 15 min of use for each of the ENDS IPs evaluated. Mean plasma nicotine concentrations declined to less than 6 ng/mL for all IPs by 60 min. Baseline-adjusted nicotine PK parameters, including nicotine uptake during the first 15 min following the start of product use (AUC_nic 0–15_), C_max_, AUC_nic0–60_, and the time to reach the maximum nicotine concentration (T_max_) across the 60-min sampling period are summarized in Table 2.

**Table 2:** Plasma nicotine pharmacokinetic parameters across Vuse Solo variants.

PK parameters assessed	Original (N = 28)	Mint (N = 34)	Tropical (N = 28)	Fusion (N = 28)
C_max_ (ng/mL) (95% CI)	6.91 (5.49–8.70)	6.53 (5.26–8.10)	8.21 (6.87–9.81)	6.67 (5.20–8.56)
AUC_nic0–15_ (ng min/mL) (95% CI)	56.19 (42.85–73.69)	63.36 (50.64–79.27)	76.28 (63.38–91.81)	56.15 (43.81–71.97)
AUC_nic0–60_ (ng min/mL) (95% CI)	223.79 (180.13–278.03)	215.37 (175.92–263.68)	263.52 (218.92–317.20)	206.87 (156.70–273.10)
T_max_* (min) (range)	11.00 (5.0–60.0)	11.00 (3.0–30.0)	11.00 (3.0–30.4)	11.00 (3.0–30.0)

PK Parameters presented in this table are baseline-adjusted geometric mean values except for T_max_.

Subjects with C_max_ < 1.0 ng/mL were excluded from this analysis.

*CI* confidence interval, *C*_*max*_ maximum nicotine concentration, *AUC*_*nic0–15*_ area under the curve for nicotine exposure over 15 min, *AUC*_*nic0–60*_ area under the curve for nicotine exposure over 60 min, *T*_*max*_ time to maximum nicotine concentration.

*T_max_ reported as median value, followed by the range for minimum and maximum.

**Figure 2:**
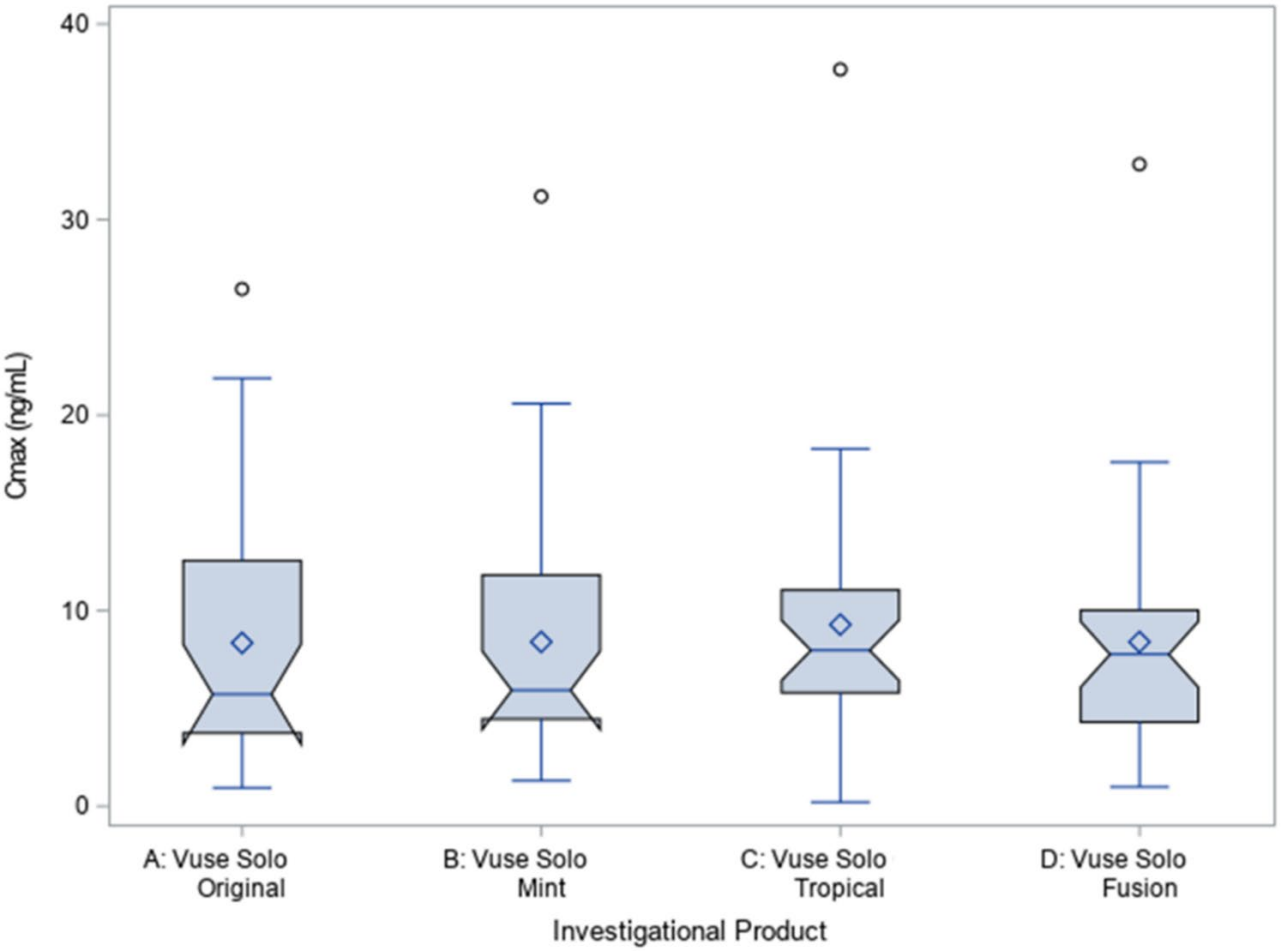
Notched box plot of baseline-adjusted C_max_ for four flavor variants of Vuse Solo.

**Figure 3:**
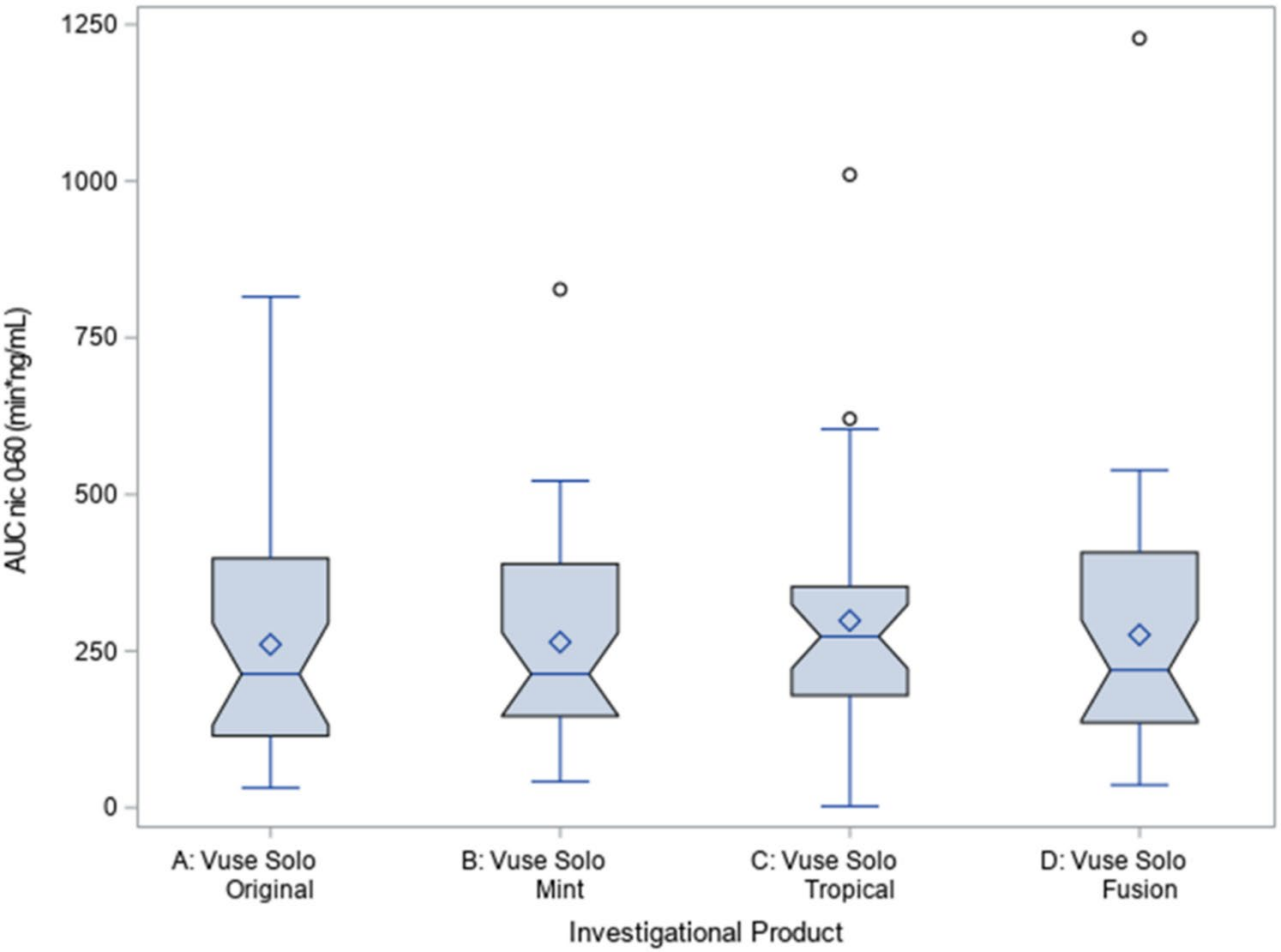
Notched box plot of baseline-adjusted AUC_nic0-60_ for four flavor variants of Vuse Solo.

This study was not designed to statistically compare flavors; the primary endpoints, C_max_ and AUC_nic0–60_, were qualitatively assessed across the ENDS flavor variants to evaluate nicotine uptake across the e-liquid flavors as shown using notched box plots of baseline-adjusted C_max_ and AUC_nic0–60_ shown in Figs. 2 and 3 respectively, to compare the distributions and point estimates in C_max_ or AUC_nic0-60_ across the flavors. The 95% CI of nicotine uptake parameters C_max_ and AUC_nic0–60_ overlap with each other among all Vuse Solo flavor variants tested.

### Product liking results

Overall product liking (OPL) was assessed at the 13-min timepoint during test sessions after product use initiation using an 11-point numeric rating scale (NRS) and results are presented in Table 3. Mean OPL scores (SD) ranged from 5.3 (2.5) for the Mint flavor to 7.5 (1.9) for the Tropical flavor and were 6.4 (2.5) and 6.0 (2.5) for the Original and Fusion flavors, respectively. Median values reflect a similar trend.

**Table 3:** Summary of overall product liking scores.

Summary statistics	Original (N = 35)	Mint (N = 39)	Tropical (N = 38)	Fusion (N = 36)
Number of subjects	30	34	29	29
OPL mean (SD)	6.4 (2.5)	5.3 (2.5)	7.5 (1.9)	6.0 (2.5)
Median	7.0	5.5	8.0	7.0

Range: 0–10. Overall Product Liking (OPL) was measured on a numerical rating scale from 0 to 10, with 0 = strongly dislike and 10 = strongly like.

*SD* standard deviation.

### Adverse events

Nine of 148 (6%) subjects experienced nine adverse events during the study. Gastroesophageal reflux (one subject) and oropharyngeal pain (one subject) were each considered by the principal investigator (PI) to be possibly related and related to IP, respectively. All other adverse events were judged by the PI not to be related to IP. All adverse events were of mild intensity except for moderate ocular hyperemia in one subject, which was deemed unrelated to product use and led to subject withdrawal from the study by the PI. No serious adverse events were reported.

## Discussion

We evaluated PK parameters of four flavor variants of e-liquids used in Vuse Solo ENDS which include 4.8% nicotine by weight (~ 57 mg/mL), and contain nicotine salts following an acute exposure in predominately ENDS naïve smokers. Our data showed that subjects achieved similar overall nicotine exposure (AUC_nic0–60_), maximum plasma nicotine concentrations (C_max_), and time to maximum concentrations (T_max_) while using one of the four flavor variants of Vuse Solo ENDS. Evaluation of PK parameters, C_max_ and AUC_nic0–60_, showed similar nicotine uptake distribution patterns across all flavors variants with overlap of the 95% confidence intervals (CIs) around the medians in both C_max_ and AUC_nic0–60_ (illustrated as the notches in boxplots, Figs. 2, 3). In addition to the PK assessments, product liking (OPL) for each flavor was assessed at the 13-min timepoint (Table 3).

As stated above, the results and distribution of baseline adjusted C_max_ were similar across flavor variants (Fig. 1). By comparison, baseline-adjusted maximum nicotine concentrations (C_max_) of Vuse Solo flavor variants (Table 2) in this study are higher than those previously reported in two abuse liability (AL) studies by Stiles et al. using Vuse Solo ENDS Original (tobacco)^16^ and Vuse Solo ENDS Menthol^17^ (for reference, the 29 mg nicotine e-liquid used in the Stiles papers corresponds to roughly the same nicotine concentrations (57 mg/mL or 4.8%) as was used in the current study). It is important to note key differences among study designs of the abuse liability studies performed by the Stiles et al. and the current study. The former studies utilized a crossover (Williams) design and were conducted in an ambulatory setting, whereas our study utilized a parallel design with a test session in confinement. The use of a parallel design is a limitation of this study because in a parallel study, as there will be larger CIs with the plasma nicotine concentrations which can increases the chance of type II errors. In addition, abuse liability studies by Stiles et al., had approximately 7 days of at-home product acclimation with instruction to use ENDS IP at least once (as did our study), but IP use compliance was not assessed. In our study, subjects were told to use the ENDS IPs as often as they liked, and product use compliance was confirmed by weighing used cartridges at the end of the at-home trial period. Thus, differences in study design as well as level of familiarity with study products and general differences between study populations may account for variability among data reported. In a study to evaluate the abuse liability of ENDS in experienced ENDS users by Hajek et al.^18^, Vuse Solo Original 4.8% was evaluated as one of the ENDS comparators and the results demonstrated that Vuse Solo Original 4.8% users achieved baseline-adjusted C_max_ of 13.6 ng/mL (9.7 SD), which is higher than what was found in the current study, but still lower than the C_max_ reported for cigarettes (17.9 ng/mL [16 SD]). In contrast, in an abuse liability study of cigarette smokers conducted by Goldenson et al.^19^, the authors reported that Vuse Solo Original 4.8% had a baseline adjusted C_max_ of 6.8 ng/mL, which was similar to our study findings, and lower than cigarette C_max_ (15.4 ng/mL). Similarly, another abuse liability study by Campbell et al., with a similar subject population as the current study, reported a mean baseline-adjusted C_max_ for Vuse Solo Original 4.8% as 5.48 ng/mL^13^. Additional results from other abuse liability studies suggest that experienced users of ENDS demonstrate higher nicotine uptake, compared to naïve ENDS users, but in general the C_max_ following acute exposure to ENDS was less than that observed with smoking a cigarette, regardless of the ENDS use experience.

As was the case with C_max_, baseline-adjusted AUC_nic0–60_ results and their distribution were also similar across Vuse Solo flavor variants. There are limitations in comparing the overall nicotine exposure, AUC_nic0-60_, between the current study against published works due to the differences in AUC calculations performed across studies based on the duration of observation^16–18^. Goldenson et al., in an AL study among cigarette smokers, reported a baseline-adjusted AUC_nic0–60_ of 216 min ng/mL for Vuse Solo Original 4.8%, which is similar to the AUC_nic0-60_ for Vuse Solo Original in this study of 223.79 min ng/mL^19^. Taken together, the PK findings from this reported study are in agreement with previously published data using Vuse Solo ENDS.

St. Helen and colleagues examined the impact of flavors (Strawberry vs. Tobacco) on nicotine uptake and topography^20,21^. The authors found no statistically significant differences in PK parameters and in puffing behavior and noted the need for further investigation. Of note, these differences were seen in subjects who used fruity or sweet flavored e-liquids in their own ENDS products, suggesting potential subject bias towards flavors that resembled their usual flavors. Our study had three “fruit/sweet” flavors, Mint, Tropical and Fusion. While Tropical flavor achieved highest C_max_ and AUC_nic0–60_, two of the fruit/sweet flavors (Mint and Fusion) resulted in lower PK parameters than non-fruity flavor (Original) (Table 2). In the context of the current study design, where ENDS naïve subjects used ENDS products ad libitum for 10 min, rather than following a puffing regiment, our data suggest that individual preference for flavor and subsequent use pattern, may be the key driver of the differences we saw in PK parameters that is supported by Gades et al.^22^.

In addition to PK assessments, subjects were also asked to rate OPL for the Vuse Solo flavor variants on an 11-point NRS at 13 min after initiating the ENDS use. Subjects rated the Tropical flavor highest, followed by the Original (tobacco), Fusion, and Mint flavor (Table 3). These reported scores, appear to be in alignment with previously published AL data using Vuse Solo. In two AL studies by Stiles et al., subjects assessed PL for two flavors (Vuse Original and Menthol) at multiple timepoints over six hours during and after start of ENDS use. In both studies by Stiles et al., maximum PL scores (E_max_) for Vuse Solo Original and Menthol (36 mg/mL nicotine) were reported at 4.13 and 4.53, respectively^16,17^, compared to 6.4 and 5.3 for OPL scores of Vuse Solo Original and Mint 4.8% (57 mg/mL nicotine) reported in this study. Similarly, in the AL studies by Goldenson et al., and Campbell et al., where Vuse Solo Original 4.8% was used as an ENDS comparator, mean maximum PL scores were between 4.5 and 5, and 5.56, respectively^19,13^. In addition, review of single OPL assessment among flavor variants in our study show a similar pattern that was observed with the PK parameters, where Tropical achieved the highest OPL, and the Original flavor scoring higher OPL than the two-remaining fruit/sweet flavor variants, Mint and Fusion, again suggesting individual preference and subsequent use pattern is the key driver of the nicotine uptake. Lastly, we measured cartridge weights before and after PK test sessions as a surrogate for e-liquid consumption and showed use of Tropical flavor was marginally higher than other flavors (Table 1). It should be noted that Topical Fruit flavor obtained both the highest mean C_max_ and the highest mean liking score; we suggest that this relationship could be followed up in future studies.

Similarly, in a topography study to assess the effect of flavor in regular smokers, Voos et al. concluded that the flavors used in their study delivered differential amounts of nicotine, potentially associated with product use topography, and that differences in subjective effects are not solely a product of nicotine delivery and recommended additional research^23^. In contrast, Cobb et al., assessed subjective effects among young adult smokers using ENDS with three flavors (cream, tropical fruit, and tobacco/menthol) at nicotine concentrations ranging from 0 to 36 mg/mL nicotine and concluded that e-liquid flavors did not appear to have significant impact on subjective effects^24^. Recent publications suggest that sufficient product appeal or product liking as well as delivery of sufficiently high amount of nicotine per use appear to be important in facilitating either reducing number of cigarettes used, or complete switching to take full advantage of reduced toxicants found in ENDS and therefore leading to tobacco harm reduction^22,25–27^. Thus, additional research is needed to determine the implication of overall product liking scores on both PK parameters and e-liquid consumption.

In this study, subjects were asked to familiarize themselves with ENDS products at home for one week prior to a PK test session. For at-home trial use, subjects were dispensed two cartridges of ENDS product flavor they were randomized to use during a PK test session with an option to request more had they used both prior to the end of the at-home trial period. Subjects were encouraged to use ENDS at least once a day while they continue to use their usual brand of cigarettes. All subjects returned one partially used cartridge at check-in, along with their second cartridge which no subject had begun to use. The amount of e-liquid consumed was measured by differences in cartridges weights obtained before and after a week of at home use, where ENDS naïve subjects were encouraged to use the ENDS ad libitum as often as possible. We found that subjects used approximately 10% of e-liquid by weight ranging from 0.16 (± 0.17) mg to 0.21 (± 0.25) mg across the four flavors (Table 1). These results were similar to e-liquid consumption observed on the first day of exclusive ENDS use in a biomarker of exposure study by Round et al.^8^. While the duration of total e-liquid consumption for our subjects were over a week versus a single day, we believe that subjects were sufficiently acclimated to ENDS IPs during their at-home trial period prior to the PK test sessions. In future studies, compliance could be increased by frequent follow up (e.g., phone call) during the familiarization period and only using weight changes in the cartridge is a limitation of the study.

Subjects were also required to abstain from tobacco products for 12 h prior to PK test sessions to ensure their blood nicotine concentrations were close to baseline prior to the start of product use. Furthermore, we collected blood samples at intervals that allowed characterization of nicotine PK following ad libitum ENDS exposure and the nicotine concentrations of blood samples were baseline-adjusted to ensure more accurate results. Lastly, for this study, subjects were allowed a 10-min ad libitum use of the ENDS^16–18,28–30^. We chose the duration to align with an estimated duration to smoke a single CC. A 5-min ENDS use duration has been used in other studies; however, subjects in these studies were given a puffing regimen (use same as a CC or 30 s between puffs)^27,31,32^. We believe 10-min ad libitum use duration provided ENDS naïve users an ample time to use ENDS and provided nicotine PK exposure profiles influenced by a user preference and ultimately conservative data that is being presented here^23,25,28,29,31–41^. In addition, had we chosen to include a puffing regimen, it is likely that the data would not have reflected the differences we saw in PK parameters that are likely driven by user preference further validating the assertion from Gades et al. that in achieving tobacco harm reduction, availability and variety of both flavor and nicotine strength are important for ENDS naïve smokers^22^. Furthermore, a recent study by Ebajemito et al., as well as data from an unpublished study indicates higher nicotine uptake in subjects during ad libitum puffing as compared to control puffing regimen over a given ENDS use duration^42^. Therefore, we are confident that our data not only represents a conservative estimates of PK parameters of nicotine from ENDS use in ENDS naïve population, but also suggests that flavors will have an impact on nicotine uptake when the only difference are the flavor in e-liquid when nicotine concentration are held at constant. In future studies, the duration of ENDS use may be modified to align with what is being reported in the literature to 5 min^23,24,29,33–36,38,42–47,48^.

This study had several limitations. Although we allowed participation of dual users, more than 90% of our subjects were exclusive cigarette smokers^49^. As the prevalence of dual and poly use of multiple types of tobacco and nicotine products, such as ENDS or other non-combustible nicotine products, continues to increase, inclusion of a greater proportion of dual users of CC and ENDS in future studies may be useful to make study findings more applicable to current nicotine and tobacco users^49–51^. Future studies may also benefit from cross-over designs to evaluate nicotine PK with multiple flavor variants to reduce inter-user variability.

In conclusion, the primary endpoints of this study, C_max_ and AUC_nic0-60_, were similar across all four flavors as evidenced by the overlap of 95% confidence intervals. Furthermore, although this study was not designed to compare between flavors, it appears that flavors are not the primary drivers in nicotine exposure in an acute exposure setting as much as individual use behavior. Future study designs could address the flavor comparisons. The results of this study may add to the growing body of literature regarding the effects of flavors on nicotine delivery and uptake.

## Methods

This study was a single-center study (ClinicalTrials.gov identifier: NCT03234010, 31/07/ 2017) designed to evaluate plasma nicotine uptake and overall product liking with use of four flavor variants of the Vuse Solo ENDS (Vuse Solo Original, Mint, Tropical and Fusion) in tobacco consumers who were exclusive smokers or dual users of cigarettes and ENDS. The study was completed at a single clinical research site (DaVita Clinical Research, Lakewood, CO) and was reviewed and approved by Chesapeake Institutional Review Board (Columbia, MD).

No formal sample size calculations were performed as the study was designed to assess nicotine uptake from flavor variants of Vuse Solo ENDS and no comparisons between flavors was intended. The study was designed to assess nicotine PK from use of flavor variants for the purpose of regulatory submission, rather than comparing a single flavor against other flavor variants. The target number of subjects to be randomized for this study was 35 subjects per flavor variants or total of 140. This allowed for approximately 15% drop out rate (5 subjects per flavor variant) with goal of obtaining 120 study completers or 30 study completers per flavor.

Attempts were made to recruit 15–20% African-Americans within each IP group in an effort to balance the study sample for the reported percentage of U.S. smokers who are African American^1^. During a screening visit, eligibility criteria were assessed to ensure that potential subjects met all criteria for inclusion and none of the exclusion criteria. Eligible subjects included male and female smokers, aged 21–60 years who self-reported smoking 10 or more cigarettes per day for at least the previous 6 months, or dual users who self-reported smoking 10 or more cigarettes per day for at least the previous 6 months, and using a nicotine-containing cig-a-like or tank-style ENDS at least weekly for at least the previous 3 months. Brief periods of abstinence longer than 30 days before screening were allowed at the discretion of the PI. Smoking history was confirmed at screening and reassessed on Study Day 1 with an expired carbon monoxide (ECO) assessment, and only subjects with ECO levels greater than 10 ppm were eligible to participate in the study. Informed consent was obtained from all subjects before any study procedures were performed.

The study was conducted in accordance with the ethical standards in the Declaration of Helsinki, applicable sections of the United States Code of Federal Regulations, and ICH E6 Good Clinical Practice guidelines.

### Study product

Each Vuse Solo ENDS consist of power unit and a cartridge. Each cartridge is a sealed unit containing 0.5 mL of e-liquid comprising 4.8% nicotine by weight (~ 57 mg/mL), and containing nicotine salts, propylene glycol, glycerin, flavorings, and water. The products were powered by a rechargeable power unit and included a heating element, microchips, and a sensor. Aerosol generation is activated by detection of a pressure differential within the ENDS product during puffing.

### Study design

The study was designed as a randomized, open-label, parallel-cohort study to assess nicotine uptake in human subjects following ENDS use. The study examined plasma nicotine PK parameters in Vuse Solo ENDS with four flavor variants: Original, Mint, Tropical and Fusion.

Subjects were given a 1-week at-home trial period to become familiar with the assigned Vuse IPs. Subjects were encouraged to use ENDS at least once a day at-home trial period while they continue to use their usual brand of cigarettes. The product use compliance during the at-home trial period was monitored by obtaining cartridge weight before the dispensing and after subject checked in to the site for overnight confinement. Subjects were randomized to specific ENDS flavors and were provided two cartridges with instructions for use. Product use compliance was checked by weighing ENDS cartridges three times before and after use to a sensitivity of 1 × 10^–4^ g before and after the at-home trial period.

On Study Day 1, subjects arrived at the study site and began their check-in procedures prior to confinement. Subjects returned ENDS IP e-liquid cartridges from the at-home trial period to the site. Eligibility criteria were reconfirmed, and those who successfully completed all check-in procedures were confined to the study site for approximately 24 h. Subjects were allowed to use their assigned IP ad libitum until the start of a mandatory 12-h abstinence from tobacco and nicotine products.

On the morning of Study Day 2, each subject was given their assigned ENDS IP for use during the PK assessment. Subjects were then allowed to use the assigned ENDS IP ad libitum for 10 min (± 10 s). Start and stop times were documented. ENDS IP cartridges were weighed three times before and after use to 1 × 10^–4^ g.

During the PK assessment, blood samples were collected and processed to plasma for nicotine measurements at the following time points relative to the start of IP use: − 5, − 0.5, 3, 5, 8, 10, 11, 12, 15, 20, 30, and 60 min. The − 0.5 min sample was used as the preferred baseline sample. Samples were collected and centrifuged within 60 min of collection. The plasma was transferred into 2 methanol pre-washed tubes and frozen prior to shipment on dry ice to the bioanalytical lab. All samples were transferred to Celerion Global Bioanalytical Services (Lincoln, NE) for nicotine quantitation using a validated liquid chromatography tandem mass spectrometry method.

In addition to blood sample collection, subjects were required to provide an OPL score at 13 min after start of IP use on a numeric scale of 0 to 10, with 0 corresponding to “strongly dislike,” 5 corresponding to “neither like nor dislike”, and 10 corresponding to “strongly like.” Each subject was assessed for adverse events and vital signs, and a symptom-driven physical examination was performed, if necessary, prior to discharge from the study to ensure subject’s safety. Upon the PI’s review of subjects’ status, if there were no new adverse events or physical examination findings to warrant further follow up, subjects were discharged from the study.

### Statistical analysis

The primary PK parameters (C_max_, AUC_nic0–60_) were summarized using descriptive statistics by IP group without inter-group comparisons. Standard summary statistics for quantitative and qualitative variables were calculated.

The pharmacokinetic (PK) analyses were performed by Nuventra Pharma Sciences, Inc. located in Durham, NC. PK parameters were derived from the baseline-adjusted plasma nicotine concentrations-time data by noncompartmental methods using Phoenix® WinNonlin® (Version 6.3; Certara USA Inc., Princeton, NJ).

AUCs were calculated using the linear trapezoidal rule. C_max_ and T_max_ were obtained directly from the baseline-adjusted plasma nicotine concentration–time data. Observed concentrations below the lower limit of quantification (LLOQ; 0.200 ng/mL) were set at half the LLOQ for data summarization and analysis. Baseline adjustment was done by estimating and subtracting pre-existing nicotine levels from observed levels, and all PK parameters were calculated from the adjusted concentrations. The amount of pre-existing nicotine was estimated by using a model that assumed that nicotine elimination followed first-order kinetics^13,14^ and a nicotine half-life of 120 min^15–17^. Each individual PK profile was examined for completeness and suitability for inclusion in the analysis. Subjects with C_max_ values less than 1.0 ng/mL were considered to be not inhaling aerosol during the 10-min period of ad libitum IP use; therefore, sensitivity analyses excluding those subjects were performed.

## Supplementary Information

Supplementary Table S1.
