# Supplementary material for: Part two: an unblinded, parallel, randomized study to assess nicotine pharmacokinetics of four Vuse Solo ENDS flavors in smokers
Source: Sci Rep. 2023 Jun 1;13:8894. doi: 10.1038/s41598-023-35439-3 (PMC10235118; doi:10.1038/s41598-023-35439-3)
Supplement: Supplementary file 1 — Supplementary Table S1. [file 41598_2023_35439_MOESM1_ESM.docx]

**Supplementary Table S1: Demographic and Baseline Characteristics**

| **Subject Characteristics** | **Original (N=35)** | **Mint (N=39)** | **Tropical (N=38)** | **Fusion (N=33)** | **Overall (N=148)** |
| --- | --- | --- | --- | --- | --- |
| **Age (years)*** | | | | | |
| Mean (SD) | 33.9 (10.3) | 34.2 (9.4) | 33.2 (8.6) | 33.7 (10.2) | 33.7 (9.5) |
| **Sex, n (%)** | | | | | |
| Male | 19 (54.3) | 22 (56.4) | 19 (50.0) | 22 (61.1) | 82 (55.4) |
| Female | 16 (45.7) | 17 (43.6) | 19 (50.0) | 14 (38.9) | 66 (44.6) |
| **Race, n (%)** | | | | | |
| Black or African American | 12 (34.3%) | 12 (30.8%) | 8 (21.1%) | 13 (36.1%) | 45 (30.4%) |
| American Indian or Alaska Native | 1 (2.9%) | 2 (5.1%) | 2 (5.3%) | 0 | 5 (3.4%) |
| Asian | 2 (5.7%) | 0 | 0 | 0 | 2 (1.4%) |
| Native Hawaiian or other Pacific Islander | 0 | 1 (2.6%) | 0 | 0 | 1 (0.7%) |
| White | 18 (51.4%) | 20 (51.3%) | 25 (65.8%) | 21 (58.3%) | 84 (56.8%) |
| Multiple | 2 (5.7%) | 4 (10.3%) | 3 (7.9%) | 2 (5.6%) | 11 (7.4%) |
| **Ethnicity, n (%)** | | | | | |
| Hispanic or Latino | 11 (31.4%) | 11 (28.2%) | 7 (18.4%) | 9 (25.0%) | 38 (25.7%) |
| Not Hispanic or Latino | 24 (68.6%) | 28 (71.8%) | 31 (81.6%) | 27 (75.0%) | 110 (74.3%) |
| **Number of years smoked** | | | | | |
| Mean (SD) | 17.99 (11.17) | 18.14 (9.97) | 17.57 (8.62) | 16.88 (10.76) | 17.65 (10.05) |
| **Number of cigarettes smoked per day** | | | | | |
| Mean (SD) | 16.1 (6.01) | 15.2 (5.36) | 15.9 (4.68) | 15.5 (5.40) | 15.7 (5.33) |
| **BMI, kg/m^2^** |  |  |  |  |  |
| Mean (SD) | 27.69 (5.86) | 26.05 (5.09) | 27.07 (6.13) | 26.74 (5.61) | 26.87 (5.65) |
| Abbreviations: BMI = body mass index, SD = standard deviation.  *Age at Informed Consent | | | | | |
